# Marker-Assisted Breeding as Next-Generation Strategy for Genetic Improvement of Productivity and Quality: Can It Be Realized in Cotton?

**DOI:** 10.1155/2011/670104

**Published:** 2011-03-20

**Authors:** N. Manikanda Boopathi, K. Thiyagu, B. Urbi, M. Santhoshkumar, A. Gopikrishnan, S. Aravind, Gat Swapnashri, R. Ravikesavan

**Affiliations:** ^1^Department of Plant Molecular Biology and Biotechnology, Tamil Nadu Agricultural University, Coimbatore 641003, India; ^2^Department of Cotton, Tamil Nadu Agricultural University, Coimbatore 641003, India

## Abstract

The dawdling development in genetic improvement of cotton with conventional breeding program is chiefly due to lack of complete knowledge on and precise manipulation of fiber productivity and quality. Naturally available cotton continues to be a resource for the upcoming breeding program, and contemporary technologies to exploit the available natural variation are outlined in this paper for further improvement of fiber. Particularly emphasis is given to application, obstacles, and perspectives of marker-assisted breeding since it appears to be more promising in manipulating novel genes that are available in the cotton germplasm. Deployment of system quantitative genetics in marker-assisted breeding program would be essential to realize its role in cotton. At the same time, role of genetic engineering and *in vitro* mutagenesis cannot be ruled out in genetic improvement of cotton.

## 1. Rationale for Genetic Improvement of Cotton Fiber

Plant trichomes exhibit number of biologically important roles including protection against biotic and abiotic factors, water absorption, secretion, alluring mechanisms, and more importantly, seed dispersal [1]. While the majority of plant trichomes are multicellular, cotton (*Gossypium *spp.) produces unicellular seed trichomes commonly called “fibers,” which are of considerable economic importance and hence make cotton as the leading cash crop in the world. Cotton remains the most miraculous fiber under the sun, even after 8000 years of its first use, and no other fiber come close to duplicating all of the desirable characteristics combined in cotton. Cotton plays important role in everyone's life from the time we dry our faces on a soft cotton towel in the morning until we slide between fresh cotton sheets at night. It provides thousands of useful products and supports millions of jobs as it moves from field to fabric. Thus, cotton has its vital role in the economic, political, and social affairs of the world. World consumption of cotton fiber is approximately 27 million metric tons per year (http://www.cotton.org/). India ranks second in global cotton production and accounts for approximately 25% of the world's total cotton area and 16% of global cotton production. The cotton industry in India has 1,543 spinning units, more than 281 composite mills, 1.72 million registered looms, and an installed capacity of 36.37 million spindles [2]. The textile industry employs 30 million people directly and is the second largest employer after agriculture and contributes 29.9% of the Indian agricultural gross domestic product [3]. Though India has the largest global area in cotton, yields of cotton are low, with an average yield of 445 kg/ha compared to the world average of 755 kg/ha. Hence, it is imperative to improve the fiber productivity by improving number of fibers per seed and reducing yield loss due to biotic and abiotic stresses [4]. Further, recent advancement in spinning technology and textile processing demands for improved fiber length, strength, and uniformity. The cotton fiber is composed of nearly pure cellulose, the largest component of plant biomass; hence it is an outstanding model for the study of cellulose biosynthesis besides plant cell wall and cell elongation [5]. In addition, compared to lignin, cellulose is easily convertible to biofuels. Thus, genetic improvement of cotton fiber and cellulose may also lead to the improvement of diverse biomass crops [1]. Therefore, it is necessary to genetically improve cotton productivity and quality since it has several economical and social applications.

## 2. Conventional Cotton Breeding: Contributions and Concerns

Modern crop varieties have been developed from plant populations that have shown genetic variability. Selection, as a systematic process, consists of identification and use of superior individuals coexisting within the population. In other words, the selection changes the genetic structure of the populations as a result of preserving superior alleles and discarding the undesirable ones [6]. Thus, crop improvement by selection primarily depends on (i) discovery and generation of genetic variability in agronomic traits and (ii) precise selection of genotypes with favourable characteristics, as a product of a recombination among superior alleles at different loci. In cases where the selection is based only upon the phenotypic values, the breeders commonly confront the problem of genotype x environment interaction [7], and they used to minimize this interaction by stratifying the selection plot or using progeny test [6]. 

The genus *Gossypium *L. has long been a focus of genetic, systematic, and breeding research. Cotton was among the first species to which the Mendelian principles were applied [8] and has a long history of improvement through breeding with sustained long-term yield gains. *Gossypium* consists of at least 45 diploid and 5 allotetraploid species. The allotetraploid cotton species, which include two commercially important cultivated species, *G. hirsutum *L. and *G. barbadense* L., were generated by A- and D-compound genomes. The best living and cultivating models of the ancestral A- and D-genome parents are *G. herbaceum *and *G. raimondii*, respectively [9]. The A-, D-, and AD genome groups have received special attention, as four different species (*G. herbaceum *(A1), *G. arboreum *(A2), *G. hirsutum *(AD1), and *G. barbadense *(AD2)) have been domesticated for their abundant seed trichomes and provide the foundation for the textile industry worldwide. Relationships among genome groups have been quantified in several studies, and the closest living relatives of the diploid genome donors to allopolyploid cotton have been identified [10]. The diploid donor of the allopolyploid A_T_ genome (where the T subscript indicates the A-genome in the tetraploid (AD) nucleus) was a species much like modern *G. arboreum *or *G. herbaceum*, whereas the allopolyploid D_T_ genome is derived from a progenitor similar to modern *G. raimondii*. These well-established relationships provide a phylogenetic framework to investigate the evolution both in terms of domesticated fiber production and polyploidy [1]. Interestingly, the A-genome species produce spinnable fiber and are cultivated on a limited scale, whereas the D-genome species do not [11]. Cytological observation indicated that synteny and colinearity are largely conserved among the five tetraploid species. Conventional breeding efforts aimed at interspecific introgression have transferred specific genes and useful traits, including stronger, longer, and finer fibers [12]. 

Despite these remarkable advances in cotton improvement during the twentieth century using traditional plant breeding approaches, yield potential has reportedly plateaued over the last 3 decades and has been hindered by complex antagonistic genetic relationships [13]. *G. barbadense* (L.) is the only 52-chromosome cultivated relative of upland cotton (*G. hirsutum*, 2*n*  =  52). It is valued for its fiber length and quality, whereas upland cotton is more valued for its high yield. While these species are hybridized easily, attempts to incorporate genes from *G. barbadense* into upland cotton have generally not achieved stable introgression of the *G. barbadense* fiber properties and have resulted in poor agronomic qualities of the progeny, distorted segregation, sterility, mote formation, and limited recombination due to incompatibility between the genomes [9]. As a consequence, it is believed that the reason for recent decline in cotton yield and fiber quality was mainly due to erosion in genetic diversity which is mainly because of repeated use of same breeding materials: *G. hirsutum*-based intraspecific cross combinations [13]. As revealed by isozymes and a range of DNA molecular markers including RAPDs, AFLPs, RFLPs, and microsatellites, the genetic diversity of widely cultivating cotton (*G. hirsutum* L.) cultivars and some of its related species had been found to be low [14]. A narrow genetic base especially among agriculturally elite types is restraining their utilization, and it is a bottleneck for cotton breeding and cultivar improvement [15]. Further, cotton fiber productivity and quality aspects are genetically and physiologically complex. Breeding for such traits is time consuming and difficult because of the polyploid nature of cotton. The paucity of information about genes that control important traits and the need for more extensive usage of diverse germplasm clearly portray the importance of new and innovative approaches. The quickly expanding knowledge on gene function and the availability of novel molecular tools are expected to offer new perspectives to solve these complex problems.

## 3. Next-Generation Breeding Strategies

To incorporate and/or manipulate novel gene(s) and thereby increasing genetic diversity, all the three contemporary approaches, *namely*, trait introgression *via* marker-assisted selection (MAS), genetic engineering, and *in vitro *mutagenesis, have advantages and disadvantages. Transgenics for improved cotton fiber quality and other value-added traits have gained much attention in private sector. Number of fiber specific promoters, foreign genes that govern several novel properties, such as colour, toughness, and thermal properties have been identified and incorporated into the cotton fiber [16, 17]. However, a practical realization of this approach in routine breeding program is yet to be demonstrated. There is also a renewed interest in chemical mutagenesis and transposon-mediated gene knock-outs as means to obtain single-gene mutants affecting phenotypes of interest, since the prospects of gene identification are high and every gene affecting a trait is potentially a target [18]. Nevertheless, very few studies have employed *in vitro* mutagenesis to test its effectiveness in improving fiber quality in cotton [19].

The limitations encountered in transgenics and mutagenesis research highlighted many challenges still faced by plant biologists in creating the next generation of designer plants. Fundamental research is still needed to elucidate biochemical and signalling pathways, as well as acquiring a better understanding of the underlying mechanisms that regulate gene expression. Gene discovery and strategies to produce the desired pattern of expression and phenotype, as well as improved transformation methodology, are yet to be elucidated. Further, at the time of this writing, the transgenic approach is feasible to engineer traits that are controlled by one or a few major genes and quantitative traits like yield which is governed by polygenes are not easily amenable through transformation. 

It is increasingly believed that naturally available cotton continues to be a resource for further development of fiber at least for a decade or until a suitable tool to manipulate fiber growth and development is described [72]. In this context, interspecific trait introgression is particularly attractive since it utilizes a broad germplasm base, can be targeted to one or more specific traits, can be modulated to include thousands of genes or even entire genomes, and is readily coupled to high-throughput MAS [73]. However, as discussed, the biological and technical challenges of introgression increase as the phyletic distance between the donor and recipient genome increases. Although introgression of genes across species boundaries is a multifarious task, it is quite desirable because the gene pools of cultivated species do not contain all of the desired alleles [73]. Hence, contemporary tools for identification and introgression of best alleles from the available cotton germplasm for genetic improvement of cotton fiber are discussed in more detail.

## 4. Introgression of Novel Alleles from Cotton Germplasm: Marker-Assisted Breeding

Currently, novel molecular tools as the molecular markers have started to demonstrate their usefulness into practical plant breeding by facilitating the identification, characterization, and manipulation of the genetic variation on important agronomic traits *via *quantitative trait loci (QTL) mapping [74]. These molecular markers have been applied to unravel many biological questions in gene mapping, population genetics, phylogenetic reconstruction, paternity testing, forensic applications, comparative and evolutionary genomics, and map-based gene cloning. Over the years, advances in molecular biology have led to the introduction of several new types of molecular markers and permit establishment of high-resolution linkage maps in crop plants. These linkage maps allow breeders to identify the markers that linked to the trait of interest and use them in marker-assisted breeding program. The main reasons supporting the utilization of molecular markers in cotton breeding programs are the 100% heritability of the markers and their lower cost [75]. Hence, molecular markers are extensively being employed in selection of traits with low heritability, identification, and introgression of complex fiber productivity and quality traits from native or exotic germplasm into elite cultivar *via *MAS [76]. In plant breeding, MAS is a relatively new concept, nevertheless the original selection concept *per se* has not changed; that is, the purpose of the selection is to search and preserve the best genotypes, but using molecular markers. At the same time, it is necessary to consider effectiveness and cost of MAS (which is greatly influenced by the marker system used) besides polymorphism, technical feasibility, and so forth. The value, ease, and cost of measurement and nature of genetic control of agronomic traits will determine the way in which molecular markers may be effectively used in a breeding program [75]. A highly saturated marker linkage map is necessary for effective MAS. Both intraspecific meiotic configuration analysis and interspecific linkage analysis indicated that the cotton genome map is ~5000 cM or larger, which is considerably longer than genomes of bread wheat (3791 cM), soybean (3159 cM), corn (1807 cM), rice (1530 cM), tomato (1472 cM), and barley (1279 cM; http://www.ncbi.nlm.nih.gov/mapview/). A highly saturated genetic map of cotton with a 5,000-cM long genome will require 3,000 DNA markers to map at an average of 1 cM density [77]. The following sections describe achievements in QTL mapping of fiber quality traits, obstacles encountered, and suitable alternatives to realize the relevance of MAS in genetic improvement of cotton for fiber productivity and quality.

## 5. Achievements

Recent years have seen significant efforts in understanding the cotton genome in its structural and functional aspects. To date, hundreds of QTLs related to various aspects of fiber quality traits have been mapped (Table 1). Published genetic mapping of fiber quality traits clearly indicated that improved productivity is not always negatively associated with quality, and therefore both can be concurrently improved [78]. Further, many QTLs for fiber-related traits have been identified in the D-subgenome of tetraploid cotton (e.g., [20, 25, 28, 44, 45, 79]) despite the fact that D-genome produce very short fibers and hence not being cultivated. This suggests that the D-genome contains important genes or regulators of fiber morphogenesis and fiber properties. 

### 5.1. Interspecific Introgression of Novel Alleles

 The strategy of crossing two superior genotypes of given species to enhance the utilization of the genetic potential was mainly due to the finding that each of the two locally adapted species contained different alleles conferring fiber productivity and quality. This is contradictory to the prevailing strategy for QTL mapping, which is to choose parental lines with maximal phenotypic divergence [80]. Linkage maps in tetraploid cotton have been most densely populated by analysis of interspecific *G. hirsutum* × *G. barbadense* due to low levels of DNA polymorphism within cotton species. Mapping populations have also been developed for *G. hirsutum* × *G. tomentosum, G. hirsutum *×* G. anomalum*, and *G. trilobum *× *G. raimondii *(Table 1). The genomic exploration of other accessions of these species or other wild tetraploid cottons may yield still additional valuable alleles. There also exists considerable variability among “race stocks” (local land races) within *G. hirsutum*, which is also well worth for further investigation [81]. Although elite × elite crosses are typical of traditional plant breeding, interspecific crosses are rarely used in cotton breeding because of numerous barriers to gene flow as discussed above [23]. Marker-assisted selection mitigates many of the problems associated with interspecific crosses [23]. The finding that the *G. hirsutum *allele is favourable at some loci and the *G. barbadense *allele at other loci shows that recombination of favourable alleles from each of these species may form novel genotypes than either of the parental species.

### 5.2. Development of High-Throughput and Efficient Marker Types

The most detailed linkage map of tetraploid cotton has been constructed by Rong et al. [36] (Table 1). This map was composed of 2,584 loci in 26 linkage groups (LGs). However, the mapping population was composed of only 57 F_2_ plants, and most of the loci were RFLPs, which is laborious and time-consuming and requires the use of radioactive isotopes. The main breakthrough of DNA-based molecular markers was driven by the invention of PCR. The first widespread markers to take full advantage of PCR technology were microsatellites or simple sequence repeats (SSRs). SSRs are often markers of choice and regarded as useful markers for map construction in cotton because (i) SSRs are simple PCR-based, codominant, informative, prompt DNA markers and amenable for automation and multiplex or high-throughput analysis [65]; (ii) on an average, there is one microsatellite in every 170 kb DNA of the cotton genome [82]; (iii) SSR markers could provide a tool for identifying loci that are subgenome A or D specific; (iv) SSRs are present in the expression sequence tags (ESTs) at a frequency of 1.7% [83], and dense consensus maps including a number of EST-based functional markers become fundamental tools for comparing LGs and QTLs derived from different pedigrees. A high-density molecular map, especially one that includes functional SSR markers associated with fiber genes, will be very important in allowing direct tagging of target genes linked to fiber quality [53]. Further, the huge EST databases that are generated by sequencing initiatives allow the “*in silico”* identification of genetic variation such as single-nucleotide polymorphisms (SNPs). Recent advances in high-throughput SNP genotyping technologies can provide high-density EST/SNP map which is essential for increasing the efficiency of QTL mapping.

### 5.3. Public Databases

 There are several genome databases that have exclusively developed to serve the international cotton research community. For example, the International Cotton Genome Initiative (ICGI; http://icgi.tamu.edu/), the Cotton Genome Database (CottonDB; http://www.cottondb.org/), the Cotton Marker Database (CMD; http://www.cottonmarker.org/), Comparative Evolutionary Genomics of Cotton (http://cottonevolution.info/), TropGENE Database (http://tropgenedb.cirad.fr/en/cotton.html), National Center for Biotechnology Information (http://ncbi.nlm.nih.gov/) for EST resources, BACMan resources at Plant Genome Mapping Laboratory (http://www.plantgenome.uga.edu/), the Cotton Diversity Database (http://cotton.agtec.uga.edu/), and so forth provide genomic, genetic, and taxonomic information including germplasm, markers, genetic and physical maps, associated traits, sequences, and bibliographic citations.

### 5.4. Functional Genomics

 As discussed, the cotton fiber is a complex biological system which is the net result of the intricate interplay of elaborate developmentally regulated pathways consisting thousands of genes. As such this has been a difficult subject to tackle using conventional approaches, especially as cotton fiber does not lend itself easily to molecular analysis. It has been only in the last two decades that researchers have begun to focus on studying the underlying developmental and cellular mechanisms that control fiber properties. Since the first report of John and Crow who had cloned the *E6 *gene through differential screening of a fiber cDNA library in 1992, several reports have shown that many genes were expressed preferentially in cotton fibers [84, 85]. Many fiber-specific genes involved in fiber cell initiation, fiber elongation, or cell wall biogenesis have been identified from the comparisons of normal (wild type) versus fiber mutants of* G. hirsutum *species. Few reports have also investigated the mechanisms and genes underlying the important developmental differences between *G. hirsutum *and *G. barbadense* [86, 87]. It is currently estimated that the cotton fiber transcriptome consists ~18000 genes in case of cultivated diploid species. The cotton fiber transcriptome in allotetraploid species is similarly estimated at ~36000 genes, and it included homeologous loci from both the A_T_ and D_T_ genomes. The high genetic complexity of the fiber transcriptome in both diploid and tetraploid species accounts for 45–50% of all the genes in the cotton genome [84]. The relationship between gene-island distribution and functional expression profiling suggests the existence of functional coupling gene clusters in the cotton genome. Xu et al. [88] identified three fiber gene-rich islands associated with fiber initiation on chromosome 5, three islands for the early to middle elongation stage on chromosome 10, three islands for the middle to late elongation stage on chromosome 14, and an island on chromosome 15 for secondary cell wall deposition. Clustering of functionally related gene clusters in the cotton genome displaying similar transcriptional regulation indicates an organizational hierarchy and its implications for the genetic enhancement of fiber quality traits. Besides, genes that are preferentially expressed in cotton fiber have been characterized, and their promoters are being used in transgenic research [17].

## 6. Obstacles

Despite the enormous above said achievements, genetic improvement of cotton faces some specific challenges because of its polyploid genome structure, the large genome size, and so forth, and they are described hereunder. 

### 6.1. Confronts with Mapping Population

Detection of QTLs is often limited by several factors such as genetic properties of QTLs, environmental effects, population size, and experimental error. Hence, it is desirable to independently confirm QTL mapping studies [89]. Such confirmation studies may involve independent populations constructed from the same parental genotypes or closely related genotypes used in the primary QTL mapping study. Sometimes, larger population sizes may also be used. Furthermore, some recent studies have proposed that QTL positions and effects should be evaluated in independent populations, because QTL mapping based on typical population sizes results in a low power of QTL detection and a large bias of QTL effects [89]. Unfortunately, due to constraints such as lack of research funding and time and possibly a lack of understanding of the need to confirm results, QTL mapping studies are rarely confirmed. Validation of “conserved” fiber quality QTLs across populations has not been conclusive, due to the fact that the majority of these QTL studies were either derived from small and mortal (F_2_ or backcross (BCs)) populations (Table 1). As compared to F_2_ or BCs, homozygous immortalized recombinant inbred lines (RILs) constitute the preferred material for QTL mapping in many crops [65]. RILs have not been widely utilized in cotton except in some cases (Table 1), mainly due to long development timelines and difficulties in production of sufficient seeds. Though there is no clear rule for the precise population size that is required for QTL analysis, it is increasingly believed that sampling limited numbers of progeny in mapping studies tends to cause the skewed distribution of QTL effects and identification of limited number of QTLs, even if many genes with equal and small effects actually control the trait [89]. Further, in several published reports, the number of LGs exceeds the gametic chromosome number (*n  *=  26), and numerous LGs are yet to be associated with specific chromosomes (Table 1) mainly due to lack of informative markers and use of small sample size. Moreover, common identities and common nomenclature have yet to be established among many LGs in the laboratory-specific maps [53]. Physical coverage of the cotton genome by these linkage maps also remains unknown. In most of the published maps the markers were not uniformly spaced over many LGs. He et al. [77] guess that these regions may be heterochromatin or gene rich. Clusters of markers with very limited recombination were frequently present which may be indicative of QTL-rich (/gene-rich) regions of cotton.

### 6.2. QTL × Environment Analysis

 Relatively large numbers of QTL were detected for fiber quality traits, and most of the detected QTL explained only less than half of the total genetic variation (Table 1). What causes the remaining genetic variation that is unexplained by QTL in large samples? One possibility is that there are many QTLs with very small effects, as assumed in classical models of quantitative genetics and these remain undetected even with very large sample sizes. Another possibility is the higher-order epistatic interactions, which are refractory to QTL mapping [89]. Further, a recurring complication in the use of QTL data is that different parental combinations and/or experiments conducted in different environments often result in identification of partly or wholly nonoverlapping sets of QTL. The majority of such differences in the QTL landscape are presumed to be due to environment sensitivity of genes [76]. Hence, proper care of including QTL **×** environment interaction analysis, which was found to be limited in the published literature, will improve the further progress of QTL mapping towards MAS.

### 6.3. Incongruence among QTL Studies

 The use of stringent statistical thresholds to infer QTL while controlling experiment-wise error rates is another reason for identification of only a small fraction of these nonoverlapping QTLs [90]. Small QTL with opposite phenotypic effects might occasionally be closely linked in coupling in early generation populations and separated only in advanced-generation populations after additional recombination. Comparison of multiple QTL mapping experiments by alignment to a common reference map offers a more complete picture of the genetic control of a trait than can be obtained in any one study [76]. However, lack of common set of anchored markers in the published reports limits the comparison of QTLs across the genetic backgrounds.

### 6.4. Complexities in Integration of Functional Genomics with QTLs

 Fiber gene function is highly conserved in the genomes of wild and cultivated species, as well as diploid and tetraploid species, despite millions of years of evolutionary history [91]. The phenotypic variation in fiber properties therefore is more likely one of quantitative differences in gene expression as opposed to differences in the genotype at the DNA level [84]. Hence, further studies are required to understand the number of copies of the genes, their regulation, and specific function in fiber development. Though systematic transcriptomic approaches can be combined with QTL analyses (discussed below), these studies do not address the occurrence of alternative splicing or the posttranslational modifications of the proteins. In addition, proteins can move in and out of other macromolecular complexes and thus modifying their functionality. This level of complexity cannot be tackled using transcriptomics alone, and hence, it is vital to include proteomics [92]. On the other hand, biochemical functions of only a small proportion of the identified proteins have been demonstrated and/or determined based on the assumptions that proteins sharing conserved domains have the same activity. Hence, the leftover proteins (domains of unknown function) remain as a challenge for elucidation of their biological function. In addition to that quantitative data on proteome and metabolome is still in its infant stage, and protein-protein interactions and protein with other macromolecules remain to be revealed. Therefore, complete knowledge on fiber growth and development at molecular level and its integration with QTL mapping is essential to design next-generation breeding strategies.

## 7. Alternatives and Future Perspectives

The importance and successful applications of MAS in crop breeding program have been shown in tomato, soybean, maize, pearl millet, rice, and so forth, [93, 94]. However, the realization of value of MAS in routine cotton breeding program for fiber productivity and quality has been realized only in few reports [17]. It highlights several insights and improvement in the current methodologies and tools, and the following strategies are proposed for successful MAS in cotton.

### 7.1. Meta Analysis of QTLs—Synergy through Networks

 Though QTLs for several common traits were mapped, direct comparisons cannot be conducted since no common markers existed among these studies. Detected QTLs are held up within family, the size of QTL effects that can be detected are limited, and inferences are restricted to a single population and set of conditions [95]. Thus, one direction for QTL analysis is to combine information from several or many studies by meta-analysis. Integration of QTL from different populations into a common map facilitates exploration of their allelic and homeologous relationships, though the level of resolution is limited by comparative marker densities, variation in recombination rates in different crosses, variation in gene densities across the genome, and other factors. Using a high-density reference genetic map which consists of 3475 loci in total, Rong et al. [76] reported alignment of 432 QTLs mapped in one diploid and 10 tetraploid interspecific cotton populations and depicted in a CMap resource. Lacape et al. [66] conducted meta-analysis of more than thousand QTLs obtained from the RIL and BC populations derived from the same parents and reported consistent meta-clusters for fiber colour, fineness, and length. As per their discussion, although their result on cotton fiber can hardly support the optimistic assumption that QTLs are accurate, they have shown that the reliability of QTL-calls and the estimated trait impact can be improved by integrating more replicates in the analysis. Hence, it is imperative to verify the regions of convergence with new maps which share common markers with the consensus map produced by Rong et al. [76] and Lacape et al. [66]. A network collaborative project was initiated recently at this university by involving four institutes that are actively engaged in cotton genetic improvement. This project provides impetus for application of molecular markers and strengthens the capacity of partner institutions in meta-analysis of identified QTLs. In contrast, the level of public-private collaborations in such analysis is almost negligible at present.

### 7.2. Map-Based Cloning

 As QTL mapping results accumulate over the next years, attention will turn to clone QTLs and then to using them. This requires higher resolution of QTL mapping, combined with a dense marker map [96]. A centimorgan (cM), corresponding to a crossover of 1%, can be a span of 10–1000 kbp and can vary across species or even within the chromosome of the given species [94]. This region may contain both desirable and undesirable genes, and hence to avoid the linkage drag of undesirable traits, it is important to establish the causal relationship between the QTL and phenotype using positional or map-based cloning. The physical size of a cM in cotton is not prohibitive to map-based cloning, but the lengthy genetic map will require a large number of markers in order to be sufficiently close to most genes for “chromosome walking” [9]. A new high-throughput marker, SNPs, is gaining its importance in this context, but huge initial investment for its generation necessitates simple innovative and economic marker techniques. It is also important to note that instead of using anonymous DNA markers, development and use of gene-specific functional markers such as SRAP, TRAP, and PAAP may increase the efficiency of map-based cloning. Further, map-based cloning in polyploids such as cotton introduces a new technical challenge not encountered in diploid (or highly diploidized) organisms, for example, that virtually all “single-copy” DNA probes occur at two or more unlinked loci. This makes it difficult to assign megabase DNA clones to their site of origin. One possible approach to this problem is the utilization of diploids in physical mapping and map-based cloning.

### 7.3. Cotton Genome Sequencing

 Decoding cotton genomes will be a foundation for improving understanding of the functional and agronomic significance of polyploidy and genome size variation within the *Gossypium* genus [1]. The whole-genome shotgun sequence of the smallest *Gossypium *genome, *G. raimondii*, provided fundamental information about gene content and organization [97]. This sequence will be used to query homologous and orthologous genomes and to investigate the gene and allele basis of phenotypic and evolutionary diversity for cotton improvement. A good parallel approach may be to search for candidates in species that are having naturally superior fiber qualities. Sequencing of G*. raimondii* genome established the critical initial template for characterizing the spectrum of diversity among the eight Gossypium genome types and three polyploid clades and provided a reference for sequencing many genomes in *Gossypium* species which is essential for further improvement of cotton.

### 7.4. Advances in Functional Genomics

 Several studies performed to compare the structural differences in the genomes have shown that the difference is in the expression pattern, rather than in the presence or absence of particular genes. The comparison of gene expression profiling between contrasting genotypes with respect to fiber quality can be extended to transcription profiling at the QTL level, and the genes identified at such QTLs may potentially be better candidates for superior fiber quality. In addition to cDNA and oligonucleotide microarrays, tiling path arrays can also be used to study gene expression in plants [98]. The advantage of tiling path arrays over conventional microarrays is that they are not stuck-up with the gene structure and hence provide unbiased and more accurate information about the transcriptome. In addition, they provide knowledge on transcriptional control at the chromosomal level. The use of tiling path arrays could help to provide better understanding on the fiber transcriptome at the genome-wide level, and it is yet to be tried in cotton. This will result into a paradigm shift from MAS to genomics-assisted selection.

### 7.5. System Quantitative Genetics—Bridging Subdisciplines

 The ultimate objective of QTL mapping is to identify the causal genes or even the causal sequence changes, the quantitative trait nucleotides (QTNs). While this remains a major challenge, it has been achieved in a few instances [99]. Identification of candidate genes and enrichment of functional markers within small targeted genomic regions are driven by the increasing availability of sequence resources, genomic databases and by technological developments. If functional candidate genes for a trait are not known, colocation of candidate gene polymorphisms with map positions, linkage to QTL, association of alleles with specific traits, or the identification of syntenic regions among genomes can help to select positional candidate genes for the trait [100]. In an another approach called genetical genomics, gene expression profiles are quantitatively assessed within a segregating population, and expression quantitative trait loci (eQTL) can be mapped like classical QTLs [66, 101]. Though global eQTL mapping studies, using whole genome microarrays, have been published in yeast, *Arabidopsis*, maize, and eucalyptus, it is in preliminary stage in cotton [66]. In addition, a comparative picture of transcript *versus* protein abundance indicates that functionally important changes in the levels of the former are not necessarily reflected in changes in the levels of the later. It also holds good for metabolomes too. Hence, genes, proteins, metabolites, and phenotypes should be considered simultaneously to unravel the complex molecular circuitry that operates within the cell. A complete elucidation of the genotype-phenotype map does not seem to be feasible unless we can include all possible causal variables in the network-inference methodology. One has to take a global perspective on life processes instead of individual components of the system. The network approach connecting all these subdisciplines indicates the emergence of a system quantitative genetics [94].

### 7.6. Association Mapping and Alternatives

 Association mapping provides another route to identifying QTLs that have effects across a broader spectrum of germplasm, if false positives that are caused by population structure can be minimized [95]. In addition, QTL mapping in biparental populations reveals only a slice of the genetic architecture for a trait because only alleles that differ between the two parental lines will segregate. Therefore, more comprehensive analyses of genetic architecture require consideration of multiple populations that represent a larger sample of the standing genetic variation in the species [95]. An important genetic resource developed in recent years is the construction of nested association mapping (NAM) population. The NAM population is a novel approach for mapping genes underlying complex traits, in which the statistical power of QTL mapping is combined with the high (potentially gene-level) chromosomal resolution of association mapping, and it has been adapted in maize [99]. Although sufficient diversity must be present in each association mapping panel, too much phenotypic diversity (or poor adaptation to any specific growing environment) may make it difficult to phenotype a panel in an association study. Thus, more region-specific association mapping panels may need to be created that contain germplasm more suited to specific growing regions. One such panel is being created at this university.

### 7.7. Improved Databases

 There is a great need to expand bioinformatic infrastructure for managing, curating, and annotating the cotton genomic sequences that will be generated in the near future. The cotton genome sequence and functional genomics database of the future should be able to host and manage cotton information resources using community-accepted genome annotation, nomenclature, and gene ontology. Some existing databases may be upgraded to effectively handle a large amount of data flow and community requests, but additional resources will be sought to support key bioinformatic needs.

## 8. Conclusion

Significant strides have been made particularly with regard to understanding the phenotypic and molecular diversity in the cotton germplasm, identification of QTLs linked to fiber productivity and quality. Yet, the application of molecular marker-assisted breeding tools to accelerate gains in cotton productivity has barely begun, and there is vast potential and need to expand the scope and impact of such innovative breeding program. Progress in this direction will be further enhanced by bringing the information generated through “omics” studies. Further, as discussed above, involvement of innovative strategies, resource pooling, capacity building to deploy marker-assisted breeding in cotton will eventually lead to develop cotton cultivars improved with improved productivity and quality.

## Figures and Tables

**Table 1:** Details on mapping population, markers, linkage maps, QTLs, and maximum phenotypic variation identified for fiber quality traits in cotton.

Species involved^a^	Population		Markers^c^	No. of mapped loci	Map length (cM)	Total linkage groups	Major QTL reported for	Chr./LG^d^	Maximum phenotypic variance observed (%)	Ref.^e^
	Type^b^	Size								
*Gh *(palmeri) × *Gb *(K101)	F_2_	57	RFLP	705	4675	41	—			[9]

*Gh (*CAMD-E) × *Gb* (Sea Island Seaberry)	F_2_	271	RFLP	261	3767	27	Fibre strength	D02	13.3	[20]
							Fibre length	D03	14.7	
							Fibre thickness	Chr.10	12.6	
							Fibre elongation	A02	14.0	
							Earliness	D04	8.1	

*Gh *(HS46) × *Gh *(MARCABUCAG8US-1-88)	F_2_ and F_3_	96	RFLP	120	865	31	Seed index	LG4	—	[21, 22]
							Micronaire	LG6	—	
							Elongation percentage	LG4	—	
							Bundle strength	LG6	—	
							2.5 per cent span length	LG3	—	
							Plant height	LG6	—	

*Gh* (Deltapine 61) × *Gb *(Sea Island Seaberry)	F_2_	180	RFLP	—	3664	26	—			[23]

*Gh *(NM24016) × *Gb *(TM1)	F_2:3_	118	RAPD and SSR	—	1058	28	—			[24]

*Gh* (MD5678ne) × *Gh* (Prema)	F_2:3_	119	RFLP	81	700.7	17	Fibre strength	LG1	35.2	[15]
							Elongation percentage	LG3	15.3	
							2.5 per cent span length	15	44.6	
							Micronaire	1	40.0	
							Uniformity ratio	7	33.6	
							Lint yield	10	9.2	
								17	15.7	

*Gh* (TM 1) × *Gb *(3-79)	F_2_	171	RFLP, RAPD, and SSR	—	4766	50	Fibre strength	Chr.25b	23.1	[25]
							Fibre length	Chr.4a	12.6	
							Micronaire	Chr.1a	43.9	

*Gh *(Siv'on) × *Gb *(F-177)	F_2_	430	RFLP	253	—	—	Seed cotton yield	Chr.14	20.5	[26]
							Boll weight	Chr.15	23.1	
							Boll number	Chr.4	17.7	
							Fiber length	Chr.6	13.7	
							Fiber length uniformity	Chr.7	13.3	
							Fiber strength	Chr.21	17.4	
							Fiber elongation	Chr.9	7.3	
							Fiber fineness	Chr.25	30.3	
							Fiber colour	Chr.11	14.9	

*Gh *× *Gh *	F_2:3_	569	RFLP	284	1502.6	47	—			[27]

*Gh *(Siv'on) × *Gb *(F-177)	F_2_ F_3_	406 208	RFLP	—	—	—	Fibre length	Chr.9	13.7	[28, 29]
							Fibre elongation	Chr.5	7.3	
							Bundle strength	Chr.1	17.4	
							Micronaire	Chr.2	30.3	

*Gh* (TM 1) × *G. anomalum *(7235)	F_2_ and F_3_	186	SSR and RAPD	—	—	—	Fibre strength	Chr.10	30.0	[30]

*Gh (*Handan208) × *Gh *(Pima90)	F_2_	129	SRAP	237	3030.7	39	—	—	—	[31]

(*Gh* (TM 1) × *Gb *(Hai7124)) × TM1	BC_1_F_1_	140	EST-SSR	624	5644.3	34	—	—	—	[32, 33]

*Gh (*Acala 44) × *Gb *(Pima S7)	F_2_	94	AFLP, SSR, and RFLP	392	3287	42	Fibre length	4	24.0	[34]
							Fibre elongation	9	42.0	
							Fiber fineness	LG35	43.0	
							Fiber tenacity	5	40.0	
							Seed weight	4	23.0	
							Number of seeds	9	22.0	

(*Gh* (Tamcot 2111) × *Gb *(Pima S6)) × Tamcot 2111	BC_3_F_2_	3662	RFLP	—	—	—	Elongation percentage	Chr.1	28.0	[35]

*Gh *(Palmeri) × *Gb *(K101)	F_2_	57	RFLP	2584	4447.9	26	—	—	—	[36]

*G. trilobum *(Skovsted) × *G. raimondii *(Ulbr)	F_2_	62	RFLP	763	1493.3	13	—	—	—	[36]

*Gh *(Acala 44) × *Gb (*Pima S-7)	F_2_	110	SSR	35	531	11	—	—	—	[37]

(*Gh *(Guazuncho 2) × *Gb *(VH8)) × Guazuncho 2	BC_1_ and BC_2_	200	SSR and RFLP	1306	5597	26	Fibre length	Chr.3	14.8	[38–40]
							Fibre strength	Chr.3	21.3	
							Elongation percentage	Chr.9	21.3	
							Micronaire	Chr.3	29.1	

*Gh* (Handan208) × *Gb* (Pima90)	F_2_ and F_2:3_	69	SSR, SRAP, RAPD, and REMAPs	1029	5472.3	26	Fibre strength	A02a	37.1	[41–43]
							Fibre length	A02	48.8	
							Micronaire	A03	28.92	
							Lint index	Chr.23	36.8	
							Seed index	Chr.6	38.5	
							Seed cotton yield	Chr.7b	39.0	
							Number of seeds per boll	Chr.14	38.1	

*Gh* (TM1) × *Gb (*Pima 3-79)	RILs	183	EST-SSR	193	1277	19 + 11 LG	Fibre length	Chr.2	5.2	[44]
							Micronaire	Chr.3	14.1	
							Bundle strength	Chr.15	5.4	
								Chr.18	6.3	
							Elongation percentage	A01	10.9	

*Gh* (7235) × *Gb *(TM-1)	F_2_ and F_2:3_	163	SSR	86	666.7	21	Fibre length	Chr.25	27.2	[45]

HS427-10 × TM-1	F_2_ and F_2:3_	169	SSR	56	557.8	17	Micronaire	Chr.25	21.8	

PD6992 × SM 3	F_2_ and F_2:3_	142	SSR	73	588	22	Fibre strength	LGD03	31.0	
							Elongation percentage	LGD03	12.6	

*Gh *(TMS22) × *G. tomentosum *(WT936)	F_2_	82	SSR	589	4259.4	52	—	—	—	[46]

*Gh* (Yumian 1) × *Gh* (T586)	F_2_ and F_2:3_	117	SSR and AFLP	70	525	20	2.5 per cent span length	Chr.6	27.4	[47]
							Fibre strength	A02/D03	20.2	
							Lint percentage	18/20	30.5	
							Elongation	Chr.5	10.9	
							Fineness	Chr.5	15.9	

*Gh* (TM 1) × *Gb* (3-79)	RILs	183	SSR	433	2126.3	46	Elongation	Chr.03	14.5	[48]
							Bundle strength	Chr.18	6.5	
							Fineness	Chr.3	17.2	
							2.5 per cent span length	Chr.18	9.0	

*Gh *(TM1) × *Gh *(7235)	RILs	258	SSR	110	810.07	22	Fiber strength	D03	26.01	[49]
							Fiber length	LG03	9.89	
							Micronaire	LG03	16.14	
							Fiber elongation	A03	5.42	
							Maturity	D03	27.06	
							Boll size	A03	8.54	
							Lint percentage	Chr.22	10.21	
							Seed index	D03	28.07	
							Bolls per plant	D03	25.52	
							Seed cotton yield	D03	15.54	
							Lint yield	D03	15.08	

*Gh *(Xiangzamian 2) × *Gh *(Zhongmiansuo I2)	RILs	180	SSR, AFLP, RAPD, and SRAP	132	865.2	26	Fibre length	D02	14.79	[50, 51]
							Bundle strength	D09	9.35	
							Micronaire	D02	15.59	
							Maturity	D02	10.75	
							Elongation	D06	9.03	

*Gh *(L-70) × *Gh* (L-47)	RILs	76	EST-SSR	—	—	—	Lint percentage	Chr.12	45.0	[52]

(*Gh *× *Gb*) × *Gh *	BC_1_F_1_	138	EST-SSR	1790	3425.8	26	—			[53]

*Gh * (7235) × *Gh* (TM-1)	RILs	207	SSR	156	1024.4	31	Fibre strength	D08	16.15	[54]
							Fibre length	D09	7.71	
							Micronaire	D08	10.91	
							Elongation percentage	LGU03	6.89	
							Seed index	D08	16.25	
							Number of bolls per plant	D08	14.16	
							Seed cotton yield	D08	20.70	
							Lint yield	D08	18.32	
							Lint percent	A03	11.40	

*Gh *(Yumian 1) × *Gh* (T586)	RILs	270	SSR	19	96.2	1	Lint percentage	6	7.90	[55, 56]
							Fiber length	6	33.36	
							Uniformity	6	25.57	
							Fiber strength	6	39.69	

*Gh *(Zhongmiansuo12) × *Gh* (8891)	RILs	180	SSR, AFLP, RAPD, and SRAP	132	865.20	26	Seed cotton yield	A09	5.10	[57]
							Lint yield	A09	4.10	
							Boll weight	D02	6.30	
							Lint percentage	A11	6.27	
							Lint index	A10	14.92	
							Seed index	D9	7.09	

*Gh *× *Gh *	Im.F_2_	171	SSR, AFLP, RAPD, and SRAPs	149	1025.1	—	Fiber length	D02	23.33	[58]
							Fiber strength	D02	7.51	
							Micronaire	A11	14.72	
							Fiber uniformity ratio	LG03	11.54	
							Short fiber index	LG04	13.74	
							Fiber elongation	D04	10.13	
							Fiber maturity	LG08	17.03	

*Gh *(CRI36) × *Gb* (Hai7124)	F_2_	186	SSR, TRAP, SRAP, and AFLP	1097	4536.7	35	—	—	—	[59]

*Gh *(Deltapine) × *Gh *(Texas 701)	F_2_	251	SSR	73	650.8	17	—	—	—	[60]

*Gh *(Handan 208) × *Gb* (Pima 90)	RILs	121	SSR	—	5472.3	26	Fiber length	A01	20.14	[61]
							Uniformity	A01	33.22	
							Maturity	Chr.14	12.04	
							Fiber strength	Chr.14	14.61	
							Elongation	Chr.10	20.54	

*Gh *(Pima 3-79) × *Gh *(NM 24016)	RILs	60	ATG-AFLP	92	—	19	—	—	—	[62]

*Gh* × *Gh *	4WC	273	SSR, EST-SSR	286	2113.3	56	Number of bolls pre plant	D02	10.20	[63]
							Seed index	D02	10.00	
							Fibre length	A11	10.3	
							Fibre strength	A10	14.00	

*Gh *(DH962) × *Gh* (Jimian5)	F_2_	137	SRAP, SSR, RAPD and RGAP	471	3070.2	51	—	—	—	[64]

*Gh *(Guazuncho 2) × *Gb* (VH8-4602)	RILs	140	SSR and AFLP	800	2044	26	Fibre length	Chr.3	—	[65, 66]
							Micronaire	Chr.15	—	
							Fiber colour	Chr.6, Chr.8, Chr.25	—	

*Gh (*HS 46) × *Gh (*MARCABUCAG8US-1-88)	RILs	188	SSR	125	965	26	Micronaire	Chr.3	13.3	[67]
							2.5 per cent span length	Chr.12	38.6	
							Elongation percentage	Chr.14	9.70	
							Bundle strength	Chr.5	13.7	

*Gh* (Yumian 1) × *Gh *(T586)	RILs	270	SSR and SRAP	604	3140.9	60	Fibre length	Chr.6	32.9	[56]
							Uniformity ratio	Chr.6	28.6	
							Bundle strength	Chr.7	31.8	
							Micronaire	Chr.7	24.7	
							Elongation percentage	Chr.7	15.1	

*Gh* × *Gb *	F_2_	214	SSR and AFLP	175	2030	42	—	—	—	[68]

(*Gh* (KC3) × *Gb *(Suvin)) × KC3	BC_1 _F_1_	62	SSR	57	911.6	19	Elongation per cent	19	13.4	[69, 70]
							2.5% span length	19	17.7	
							Micronaire	25	14.7	

*Gh *(MD17) ×* Gh* (181)	F_2_	162	SSR, SNP	132	1003	33	Lint percentage	26	87.1	[71]

*Gh *(MD17) ×* Gh* (FM966)	F_2_	100	SSR, SNP	115	779	29	Micronaire	Chr.2	12.4	
							2.5% span length	Chr.10	15.7	
							Fiber strength	Chr.16	11.1	

^a^ *Gh: G. hirsutum; Gb: G. barbadense.* ^b^F_2_: F_2_ progenies; BC: Backcross progenies; RILs: recombinant inbred lines; Im.F_2_: immortalized F_2_ population; 4WC: four way cross populations; ^c^RFLP: restriction fragment length polymorphism; SSR: simple sequence repeats; RAPD: randomly amplified polymorphic DNA; SRAP: sequence-related amplified polymorphism; EST-SSR: expressed sequence tags derived SSR; AFLP: amplified fragment length polymorphism; REMAPs: retrotransposons-based microsatellite amplified polymorphisms; TRAP: target region amplified polymorphism; ATG-AFLP: ATG sequence-specific AFLP; RGAP: resistance gene analogues polymorphism; SNP: single-nucleotide polymorphism. ^d^Chr./LG: chromosome number or linkage group.^e^Ref.: reference(s). (—): data not available.

## References

[1] Chen ZJ, Scheffler BE, Dennis E (2007). Toward sequencing cotton (*Gossypium*) genomes. *Plant Physiology*.

[2] Kambhampati U, Morse S, Bennett R, Ismael Y (2005). Perceptions of the impacts of genetically modified cotton varieties: a case study of the cotton industry in Gujarat, India. *AgBioForum*.

[3] Barwale RB, Gadwal VR, Zehr U, Zehr B (2004). Prospects for Bt cotton technology in India. *AgBioForum*.

[4] Wilkins TA, Rajasekaran K, Anderson DM (2000). Cotton biotechnology. *Critical Reviews in Plant Sciences*.

[5] Kim HJ, Triplett BA (2004). Cotton fiber germin-like protein. I. Molecular cloning and gene expression. *Planta*.

[6] Budak H, Bölek Y, Dokuyucu T, Akkaya A (2004). Potential uses of molecular markers in crop improvement. *KSU Journal of Science and Engineering*.

[7] Stromberg LD, Dudley JW, Rufener GK (1994). Comparing conventional early generation selection with molecular marker assisted selection in maize. *Crop Science*.

[8] Balls WL (1906). Studies in Egyptian cotton. *Yearbook Khediv. Agriculture Society for 1906*.

[9] Reinisch AJ, Dong JM, Brubaker CL, Stelly DM, Wendel JF, Paterson AH (1994). A detailed RFLP map of cotton, *Gossypium hirsutum* × *Gossypium barbadense*: chromosome organization and evolution in a disomic polyploid genome. *Genetics*.

[10] Udall JA, Flagel LE, Cheung F (2007). Spotted cotton oligonucleotide microarrays for gene expression analysis. *BMC Genomics*.

[11] Applequist WL, Cronn R, Wendel JF (2001). Comparative development of fiber in wild and cultivated cotton. *Evolution and Development*.

[12] Bi IV, Maquet A, Baudoin JP, Du Jardin P, Jacquemin JM, Mergeai G (1999). Breeding for ’low-gossypol seed and high-gossypol plants’ in upland cotton. Analysis of tri-species hybrids and backcross progenies using AFLPs and mapped RFLPs. *Theoretical and Applied Genetics*.

[13] Meredith WR Continued progress for breeding for yield in the USA.

[14] Lacape JM, Dessauw D, Rajab M, Noyer JL, Hau B (2007). Microsatellite diversity in tetraploid *Gossypium* germplasm: assembling a highly informative genotyping set of cotton SSRs. *Molecular Breeding*.

[15] Ulloa M, Meredith WR (2000). Genetic linkage map and QTL analysis of agronomic and fibre quality traits in an intraspecific population. *Journal of Cotton Science*.

[16] Rossbach V, Patanathabutr P, Wichitwechkarn J (2003). Copying and manipulating nature: innovation for textile material. *Fibers and Polymers*.

[17] Guo W, Sun J, Zhang T (2003). Gene cloning and molecular breeding to improve fiber qualities in cotton. *Chinese Science Bulletin*.

[18] Nadeau JH, Frankel WN (2000). The roads from phenotypic variation to gene discovery: mutagenesis versus QTLs. *Nature Genetics*.

[19] Herring AD, Auld DL, Ethridge MD (2004). Inheritance of fiber quality and lint yield in a chemically mutated population of cotton. *Euphytica*.

[20] Jiang CX, Wright RJ, El-Zik KM, Paterson AH (1998). Polyploid formation created unique avenues for response to selection in *Gossypium* (cotton). *Proceedings of the National Academy of Sciences of the United States of America*.

[21] Shappley ZW, Jenkins JN, Zhu J, McCarty JC (1998). Quantitative trait loci associated with agronomic and fiber traits of upland cotton. *Journal of Cotton Science*.

[22] Shappley ZW, Jenkins JN, Meredith WR, McCarty JC (1998). An RFLP linkage map of Upland cotton, *Gossypium hirsutum* L. *Theoretical and Applied Genetics*.

[23] Jiang CX, Chee PW, Draye X, Morrell PL, Smith CW, Paterson AH (2000). Multilocus interactions restrict gene introgression in interspecific populations of polyploid *Gossypium* (cotton). *Evolution*.

[24] Ulloa M, Cantrell RG, Percy RG, Zeiger E, Lu Z (2000). QTL analysis of stomatal conductance and relationship to lint yield in an interspecific cotton. *Journal of Cotton Science*.

[25] Kohel RJ, Yu J, Park YH, Lazo GR (2001). Molecular mapping and characterization of traits controlling fiber quality in cotton. *Euphytica*.

[26] Saranga Y, Menz M, Jiang CX, Wright RJ, Yakir D, Paterson AH (2001). Genomic dissection of genotype x environment interactions conferring adaptation of cotton to arid conditions. *Genome Research*.

[27] Ulloa M, Meredith WR, Shappley ZW, Kahler AL (2002). RFLP genetic linkage maps from four F_2:3_ populations and a joinmap of *Gossypium hirsutum* L. *Theoretical and Applied Genetics*.

[28] Paterson AH, Saranga Y, Menz M, Jiang CX, Wright RJ (2003). QTL analysis of genotype × environment interactions affecting cotton fiber quality. *Theoretical and Applied Genetics*.

[29] Saranga Y, Jiang CX, Wright RJ, Yakir D, Paterson AH (2004). Genetic dissection of cotton physiological responses to arid conditions and their inter-relationships with productivity. *Plant, Cell and Environment*.

[30] Zhang T, Yuan Y, Yu J, Guo W, Kohel RJ (2003). Molecular tagging of a major QTL for fiber strength in Upland cotton and its marker-assisted selection. *Theoretical and Applied Genetics*.

[31] Lin Z, Zhang X, Nie Y, He D, Wu M (2003). Construction of a genetic linkage map for cotton based on SRAP. *Chinese Science Bulletin*.

[32] Han ZG, Guo WZ, Song XL, Zhang TZ (2004). Genetic mapping of EST-derived microsatellites from the diploid *Gossypium arboreum* in allotetraploid cotton. *Molecular Genetics and Genomics*.

[33] Han Z, Wang C, Song X (2006). Characteristics, development and mapping of *Gossypium hirsutum* derived EST-SSRs in allotetraploid cotton. *Theoretical and Applied Genetics*.

[34] Mei M, Syed NH, Gao W (2004). Genetic mapping and QTL analysis of fiber-related traits in cotton (*Gossypium*). *Theoretical and Applied Genetics*.

[35] Chee P, Draye X, Jiang CX (2005). Molecular dissection of interspecific variation between *Gossypium hirsutum* and *Gossypium barbadense* (cotton) by a backcross-self approach: I. Fiber elongation. *Theoretical and Applied Genetics*.

[36] Rong J, Abbey C, Bowers JE (2004). A 3347-locus genetic recombination map of sequence-tagged sites reveals features of genome organization, transmission and evolution of cotton (*Gossypium*). *Genetics*.

[37] Bolek Y, El-Zik KM, Pepper AE (2005). Mapping of verticillium wilt resistance genes in cotton. *Plant Science*.

[38] Lacape JM, Nguyen TB, Thibivilliers S (2003). A combined RFLP-SSR-AFLP map of tetraploid cotton based on a *Gossypium hirsutum* × *Gossypium barbadense* backcross population. *Genome*.

[39] Nguyen TB, Giband M, Brottier P, Risterucci AM, Lacape JM (2004). Wide coverage of the tetraploid cotton genome using newly developed microsatellite markers. *Theoretical and Applied Genetics*.

[40] Lacape JM, Nguyen TB, Courtois B (2005). QTL analysis of cotton fiber quality using multiple *Gossypium hirsutum* × *Gossypium barbadense* backcross generations. *Crop Science*.

[41] Lin Z, He D, Zhang X (2005). Linkage map construction and mapping QTL for cotton fibre quality using SRAP, SSR and RAPD. *Plant Breeding*.

[42] He DH, Lin ZX, Zhang XL (2005). Mapping QTLs of traits contributing to yield and analysis of genetic effects in tetraploid cotton. *Euphytica*.

[43] He DH, Lin ZX, Zhang XL (2007). QTL mapping for economic traits based on a dense genetic map of cotton with PCR-based markers using the interspecific cross of *Gossypium hirsutum* × *Gossypium barbadense*. *Euphytica*.

[44] Park YH, Alabady MS, Ulloa M (2005). Genetic mapping of new cotton fiber loci using EST-derived microsatellites in an interspecific recombinant inbred line cotton population. *Molecular Genetics and Genomics*.

[45] Shen X, Guo W, Zhu X (2005). Molecular mapping of QTLs for fiber qualities in three diverse lines in upland cotton using SSR markers. *Molecular Breeding*.

[46] Waghmare VN, Rong J, Rogers CJ, Pierce GJ, Wendel JF, Paterson AH (2005). Genetic mapping of a cross between *Gossypium hirsutum* (cotton) and the Hawaiian endemic, *Gossypium tomentosum*. *Theoretical and Applied Genetics*.

[47] Zhang ZS, Xiao YH, Luo M (2005). Construction of a genetic linkage map and QTL analysis of fiber-related traits in upland cotton (*Gossypium hirsutum* L.). *Euphytica*.

[48] Frelichowski JE, Palmer MB, Main D (2006). Cotton genome mapping with new microsatellites from Acala ‘Maxxa’ BAC-ends. *Molecular Genetics and Genomics*.

[49] Shen X, Zhang T, Guo W, Zhu X, Zhang X (2006). Mapping fiber and yield QTLs with main, epistatic, and QTL x environment interaction effects in recombinant inbred lines of Upland cotton. *Crop Science*.

[50] Wang B, Guo W, Zhu X, Wu Y, Huang N, Zhang T (2007). QTL mapping of yield and yield components for elite hybrid derived-RILs in upland cotton. *Journal of Genetics and Genomics*.

[51] Wang B, Guo W, Zhu X, Wu Y, Huang N, Zhang T (2006). QTL mapping of fiber quality in an elite hybrid derived-RIL population of upland cotton. *Euphytica*.

[52] Abdurakhmonov IY, Buriev ZT, Saha S (2007). Microsatellite markers associated with lint percentage trait in cotton, *Gossypium hirsutum*. *Euphytica*.

[53] Guo W, Cai P, Wang C (2007). A microsatellite-based, gene-rich linkage map reveals genome structure, function and evolution in *Gossypium*. *Genetics*.

[54] Shen X, Guo W, Lu Q, Zhu X, Yuan Y, Zhang T (2007). Genetic mapping of quantitative trait loci for fiber quality and yield trait by RIL approach in upland cotton. *Euphytica*.

[55] Wan Q, Zhang Z, Hu M (2007). T_1_ locus in cotton is the candidate gene affecting lint percentage, fiber quality and spiny bollworm (*Earias* spp.) resistance. *Euphytica*.

[56] Zhang ZS, Hu MC, Zhang J (2009). Construction of a comprehensive PCR-based marker linkage map and QTL mapping for fiber quality traits in upland cotton (*Gossypium hirsutum* L.). *Molecular Breeding*.

[57] Wang BH, Wu YT, Huang NT, Zhu XF, Guo WZ, Zhang TZ (2006). QTL mapping for plant architecture traits in upland cotton using RILs and SSR markers. *Acta Genetica Sinica*.

[58] Wang B, Wu Y, Guo W, Zhu X, Huang N, Zhang T (2007). QTL analysis and epistasis effects dissection of fiber qualities in an elite cotton hybrid grown in second generation. *Crop Science*.

[59] Yu J, Yu S, Lu C (2007). High-density linkage map of cultivated allotetraploid cotton based on SSR, TRAP, SRAP and AFLP markers. *Journal of Integrative Plant Biology*.

[60] Guo Y, McCarty JC, Jenkins JN, Saha S (2008). QTLs for node of first fruiting branch in a cross of an upland cotton, *Gossypium hirsutum* L., cultivar with primitive accession Texas 701. *Euphytica*.

[61] He DH, Lin ZX, Zhang XL (2008). Dissection of genetic variance of fibre quality in advanced generations from an interspecific cross of *Gossypium hirsutum* and *G. barbadense*. *Plant Breeding*.

[62] Lu Y, Curtiss J, Miranda D, Hughs E, Zhang J (2008). ATG-anchored AFLP (ATG-AFLP) analysis in cotton. *Plant Cell Reports*.

[63] Qin H, Guo W, Zhang YM, Zhang T (2008). QTL mapping of yield and fiber traits based on a four-way cross population in *Gossypium hirsutum* L. *Theoretical and Applied Genetics*.

[64] Lin Z, Zhang Y, Zhang X, Guo X (2009). A high-density integrative linkage map for *Gossypium hirsutum*. *Euphytica*.

[65] Lacape JM, Jacobs J, Arioli T (2009). A new interspecific, *Gossypium hirsutum* × *G. barbadense*, RIL population: towards a unified consensus linkage map of tetraploid cotton. *Theoretical and Applied Genetics*.

[66] Lacape JM, Llewellyn D, Jacobs J (2010). Meta-analysis of cotton fiber quality QTLs across diverse environments in a *Gossypium hirsutum* × *G. barbadense* RIL population. *BMC Plant Biology*.

[67] Wu J, Gutierrez OA, Jenkins JN, McCarty JC, Zhu J (2009). Quantitative analysis and QTL mapping for agronomic and fiber traits in an RI population of upland cotton. *Euphytica*.

[68] Yang XL, Wang ZW, Zhang GY, Pan YX, Wu LQ, Li ZK (2009). Construction of molecular genetic map and QTL analysis of fibre quality traits in cotton. *Acta Agronomica Sinica*.

[69] Santoshkumar M (2010). *Genetic and Quantitative trait loci analysis for yield and fibre quality traits in cotton (*Gossypium* spp.)*.

[70] Urbi B (2010). *Quantitative trait loci analysis for fibre quality traits in cotton (*Gossypium* spp.) using F_2:3_ population*.

[71] An C, Jenkins JN, Wu J, Guo Y, McCarty JC (2010). Use of fiber and fuzz mutants to detect QTL for yield components, seed, and fiber traits of upland cotton. *Euphytica*.

[72] Boopathi NM, Gopikrishnan A, Jagadeesh Selvam N (2008). Genetic diversity assessment of *G. barbadense* accessions to widen cotton (*Gossypium* spp.) gene pool for improved fibre quality. *Journal of Cotton Research and Development*.

[73] Saha S, Jenkins JN, Wu J (2006). Effects of chromosome-specific introgression in upland cotton on fiber and agronomic traits. *Genetics*.

[74] Sorrells ME, Wilson WA (1997). Direct classification and selection of superior alleles for crop improvement. *Crop Science*.

[75] Winter P, Kahl G (1995). Molecular marker technologies for plant improvement. *World Journal of Microbiology & Biotechnology*.

[76] Rong J, Feltus FA, Waghmare VN (2007). Meta-analysis of polyploid cotton QTL shows unequal contributions of subgenomes to a complex network of genes and gene clusters implicated in lint fiber development. *Genetics*.

[77] He DH, Lin ZX, Zhang XL (2007). QTL mapping for economic traits based on a dense genetic map of cotton with PCR-based markers using the interspecific cross of *Gossypium hirsutum* × *Gossypium barbadense*. *Euphytica*.

[78] Paterson AH, Saranga Y, Menz M, Jiang CX, Wright RJ (2003). QTL analysis of genotype × environment interactions affecting cotton fiber quality. *Theoretical and Applied Genetics*.

[79] Ulloa M, Saha S, Jenkins JN, Meredith WR, McCarty JC, Stelly DM (2005). Chromosomal assignment of RFLP linkage groups harboring important QTLs on an intraspecific cotton (*Gossypium hirsutum* L.) joinmap. *Journal of Heredity*.

[80] Lander ES, Botstein S (1989). Mapping mendelian factors underlying quantitative traits using RFLP linkage maps. *Genetics*.

[81] Rosenow DT, Quisenberry JE, Wendt CW, Clark LE (1983). Drought tolerant sorghum and cotton germplasm. *Agricultural Water Management*.

[82] Zhao X-P, Si Y, Hanson RE (1998). Dispersed repetitive DNA has spread to new genomes since polyploid formation in cotton. *Genome Research*.

[83] Reddy OUK, Pepper AE, Abdurakhmonov I (2001). New dinucleotide and trinucleotide microsatellite marker resources for cotton genome research. *Journal of Cotton Science*.

[84] Wilkins TA, Arpat AB (2005). The cotton fiber transcriptome. *Physiologia Plantarum*.

[85] Boopathi NM, Ravikesavan R (2009). Emerging trends in enhancement of cotton fiber productivity and quality using functional genomics tools. *Biotechnology and Molecular Biology Review*.

[86] Ruan YL, Llewellyn DJ, Furbank RT (2001). The control of single-celled cotton fiber elongation by developmentally reversible gating of plasmodesmata and coordinated expression of sucrose and K^+^ transporters and expansin. *Plant Cell*.

[87] Wu Y, Rozenfeld S, Defferrard A (2005). Cycloheximide treatment of cotton ovules alters the abundance of specific classes of mRNAs and generates novel ESTs for microarray expression profiling. *Molecular Genetics and Genomics*.

[88] Xu Z, Kohel RJ, Song G (2008). Gene-rich islands for fiber development in the cotton genome. *Genomics*.

[89] Collard BCY, Jahufer MZZ, Brouwer JB, Pang ECK (2005). An introduction to markers, quantitative trait loci (QTL) mapping and marker-assisted selection for crop improvement: the basic concepts. *Euphytica*.

[90] Churchill GA, Doerge RW (1994). Empirical threshold values for quantitative trait mapping. *Genetics*.

[91] Cedroni ML, Cronn RC, Adams KL, Wilkins TA, Wendel JF (2003). Evolution and expression of MYB genes in diploid and polyploid cotton. *Plant Molecular Biology*.

[92] Jamet E, Canut H, Boudart G, Pont-Lezica RF (2006). Cell wall proteins: a new insight through proteomics. *Trends in Plant Science*.

[93] Babu R, Nair SK, Prasanna BM, Gupta HS (2004). Integrating marker-assisted selection in crop breeding—prospects and challenges. *Current Science*.

[94] Narain P (2010). Quantitative genetics: past and present. *Molecular Breeding*.

[95] Collard BCY, Mackill DJ (2008). Marker-assisted selection: an approach for precision plant breeding in the twenty-first century. *Philosophical Transactions of the Royal Society B*.

[96] Haley CS, Andersson L, Dear PH (1997). Linkage mapping of quantitative trait loci in plants and animals. *Genome Mapping: A Practical Approach*.

[97] Lin L, Pierce GJ, Bowers JE (2010). A draft physical map of a D-genome cotton species (*Gossypium raimondii*). *BMC Genomics*.

[98] Vij S, Tyagi AK (2007). Emerging trends in the functional genomics of the abiotic stress response in crop plants: review article. *Plant Biotechnology Journal*.

[99] Prasanna BM, Pixley K, Warburton ML, Xie CX (2010). Molecular marker-assisted breeding options for maize improvement in Asia. *Molecular Breeding*.

[100] Salentijn EMJ, Pereira A, Angenent GC (2007). Plant translational genomics: from model species to crops. *Molecular Breeding*.

[101] Jansen RC, Nap JP (2001). Genetical genomics: the added value from segregation. *Trends in Genetics*.

